# From evidence to care delivery: Opportunities and risks in health and science policy. A position paper of the Association of the Scientific Medical Societies in Germany (AWMF)

**DOI:** 10.3205/000336

**Published:** 2024-11-06

**Authors:** Bernhard Wörmann, Ina Kopp, Monika Nothacker, Ernst Klar, Martin Sedlmayr, Michael Vogeser, Henning Schliephake, Rolf-Detlef Treede

**Affiliations:** 1 Division of Hematology, Oncology and Tumor Immunology, Department of Medicine, Charité University Medicine Berlin, Germany; 2 AWMF Institute for Medical Knowledge Management (AWMF-IMWi), Marburg/Berlin, Germany; 3 AWMF Ad Hoc Committee on the Assessment of Medicinal Products, Berlin, Germany; 4 Institute for Medical Informatics and Biometry, Carl Gustav Carus Faculty of Medicine, Technical University of Dresden, Germany; 5 Institute of Laboratory Medicine, Hospital of the University of Munich (LMU), Munich, Germany; 6 Department of Oral and Maxillofacial Surgery, Georg August University Göttingen, Germany; 7 Department of Neurophysiology, Mannheim Center for Translational Neuroscience (MCTN), Heidelberg University, Heidelberg, Germany; 8 Department of Psychiatry and Psychotherapy, Central Institute for Mental Health, Medical Faculty Mannheim, Heidelberg University, Mannheim, Germany

**Keywords:** clinical practice guidelines, care delivery, evidence-based medicine, medical device regulation, point-of-care, guideline registries

## Abstract

**Objective:**

After over 25 years of developing clinical practice guidelines, the Association of the Scientific Medical Societies in Germany (AWMF) held a symposium to discuss the following topics in order to improve the way evidence is implemented in the delivery of care: expansion of the data pool for guideline development, the regulatory policy framework for this expansion, the transfer of clinical practice guideline statements to medical practice, the associated opportunities and risks resulting from the European legislation.

**Methods:**

The AWMF held its Berlin Forum on 27 April 2022 where experts from scientific medical societies and national institutions in the healthcare sector reported their experiences and perceptions on the topics mentioned. Three writing groups compiled the key statements from these contributions to and discussions made at the Berlin Forum into a position paper.

**Results:**

The AWMF recommends the following:

– The creation of a digital infrastructure that serves the quality assurance of clinical practice guidelines and makes their content available at the bedside and during consultations

– An increase in the number of industry-independent clinical trials on prevention, diagnostics and therapy with medicinal products, medical devices or other procedures

– The funding of registry structures to generate point-of-care healthcare data

– The reduction of excessive bureaucratic hurdles at both the national and EU level

**Conclusions:**

By making concrete recommendations in this position paper, the AWMF maps out the steps required to improve the translation of evidence to the delivery of clinical care.

## Zusammenfassung

**Fragestellung:**

Nach über 25 Jahren der Leitlinienentwicklung hat die Arbeitsgemeinschaft der Wissenschaftlichen Medizinischen Fachgesellschaften (AWMF) in einem Symposium die folgenden Themen zur Verbesserung der Implementierung von Evidenz in die Versorgung diskutiert: Verbreiterung der Datenbasis für die Leitlinienentwicklung, regulatorische Rahmenbedingungen für diese Erweiterung, Transfer der Leitlinienaussagen in die medizinische Praxis sowie Chancen und Risiken, die sich hierfür aus der europäischen Gesetzgebung ergeben.

**Methoden:**

Auf dem Berliner Forum der AWMF am 27.04.2022 trugen Fachleute aus wissenschaftlichen Fachgesellschaften und nationalen Institutionen des Gesundheitswesens ihre Erfahrungen und Vorstellungen zu den o.g. Themen vor. Hieraus und aus den Diskussionsbeiträgen beim Berliner Forum trugen drei Schreibgruppen die wesentlichen Aussagen für diesen Artikel zusammen.

**Ergebnisse:**

Die AWMF empfiehlt:

– Schaffung einer digitalen Infrastruktur für die Qualitätssicherung von Leitlinien und für die Verfügbarkeit von deren Inhalten am Krankenbett und bei der Konsultation

– Ausweitung industrieunabhängiger klinischer Studien zu Prävention, Diagnostik und Therapie mit Medikamenten, Medizinprodukten oder anderen Verfahren

– Förderung von Registerstrukturen zur Erzeugung von versorgungsnahen Daten in der Gesundheitsversorgung

– Reduktion übertriebener bürokratischer Hürden auf nationaler und EU-Ebene

**Schlussfolgerungen:**

Mit den konkreten Empfehlungen dieses Positionspapiers weist die AWMF auf notwendige Schritte zur Verbesserung der Translation von der Evidenz in die klinische Versorgung hin.

## 1 Introduction

Upon recommendation by the Council of Experts for the Concerted Action in Healthcare (KAiG), the Association of the Scientific Medical Societies in Germany (AWMF) has been coordinating the development and updating of clinical practice guidelines for Germany since 1996 [1]. Thereby, its members make a seminal contribution to the implementation of evidence-based medicine. The processes involved in the development of clinical practice guidelines and their publication for the professional and lay communities have meanwhile become standardized to a high level. This method allows the AWMF to inform health policy makers efficiently and comprehensively with well-substantiated facts as a basis for decision-making. This groundwork has proven its worth, especially during the COVID-19 pandemic. 

The AWMF has published policy demands relating to the use of evidence-based medicine as a foundation for a scientifically informed health policy (see Table 1 (Tab. 1)), [2], [3]. The drafting of concrete actionable instructions on how to optimally generate high-quality health data and practically implement such data in patient care were the topics discussed at an AWMF Berlin Forum. The core emphasis of the discussion among the experts from medicine and health policy was on the sustainable support of clinical practice guidelines as the foundation for a scientifically informed healthcare policy. This includes improving the policy framework for health research and research transfer in Germany itself [4] and a proactive monitoring of the policy frameworks and trends within the European Union.

## 2 Methods

At the Berlin Forum held by the AWMF on 27 April 2022, experts from the AWMF member societies and healthcare institutions reported on the status quo, addressing the strengths and weaknesses of national and European health research policy frameworks and how these significantly impact the translation of clinical guidelines into clinical practice. These topics were discussed with representatives of the relevant Scientific Medical Societies and other organizations in the healthcare sector. Afterwards, a paper on the contents and the derived recommendations was drafted by three writing groups. Next, the draft was subjected to multiple reviews by the participating speakers, comprehensively discussed within the AWMF Board and a consensus was reached on the final version (Table 2 (Tab. 2)).

## 3 Policy framework for health research and research transfer in Germany

One decisive requirement for the transfer of research results to the delivery of medical care is the existence and/or creation of a suitable policy framework that allows clinical trials to be conducted independently. This was the first of the three areas the 2022 Berlin Forum focused on, with the discussion thereof being chaired by Prof. Rolf-Detlef Treede, M.D.

### 3.1 Medicinal product supply

**Figure 1:**
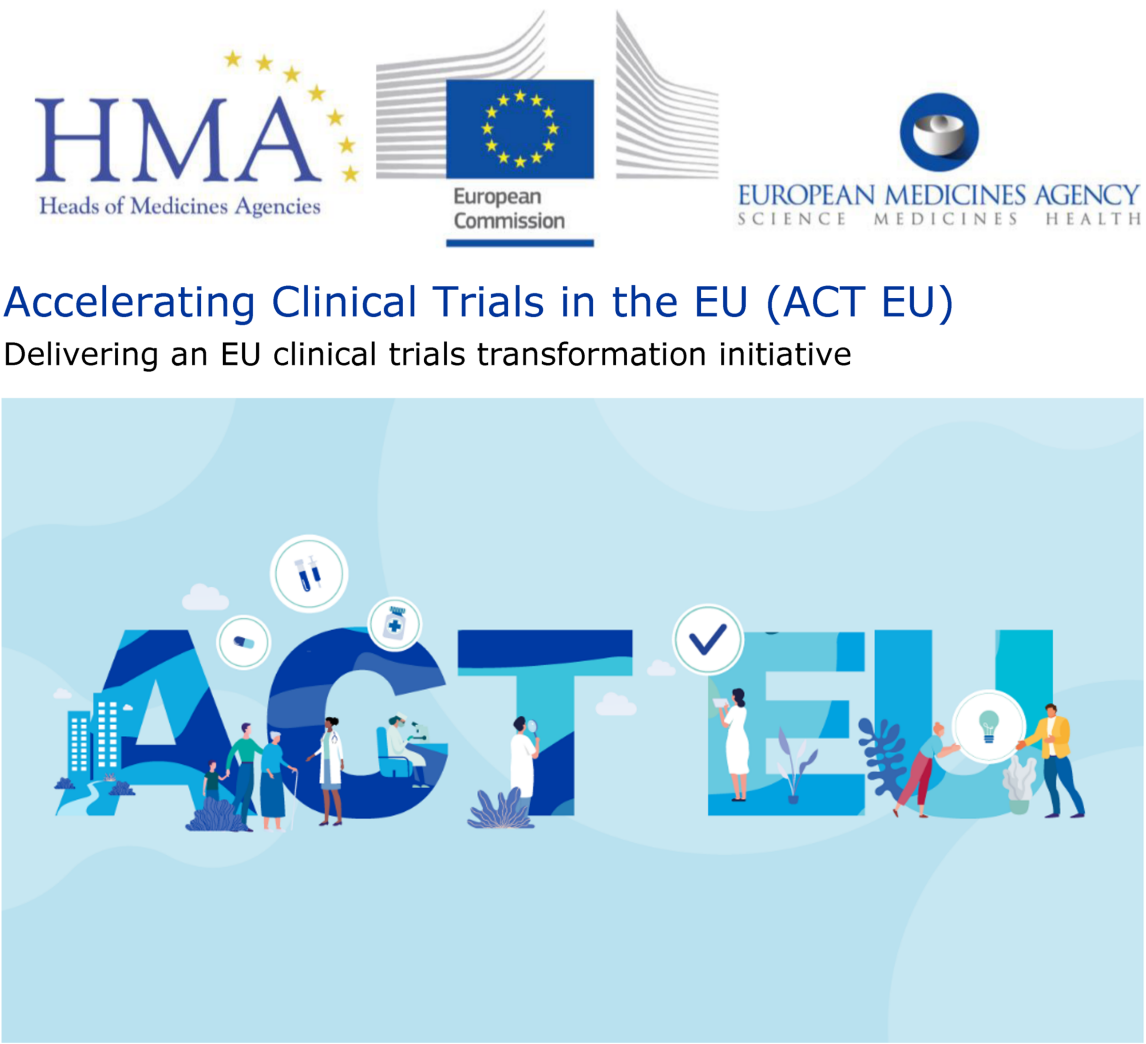
Policy framework and future trends in the European context The ACT-EU project is intended to accelerate clinical trials in the European Union. The elements mentioned therein should also be incorporated into German legislation. The ACT EU 2022–2026 work plan contains the planned project results and time sequences for the period from 2022 to 2026. Among others, the following project results are planned for 2023: establishing a process to support academic sponsors in conducting large multinational clinical trials, establishing a platform for dialogue between the various stakeholders in the clinical trials that should involve patients, health care professionals and contributors from academia, and promoting innovations in the methodology of decentralised clinical trials. Figure composed of heading and title of [24] (copyright: Heads of Medicines Agencies, European Commission and European Medicines Agency) and the image “Act EU” from [25] (copyright: European Medicines Agency)

The German Federal Institute for Drugs and Medical Devices (BfArM) plays an instrumental role at the intersection between European authorisation procedures and national care delivery. The current core areas and challenges are:

- **Clinical trials – activities based in Europe:** The legislative basis for funding high-quality clinical trials in the EU is the **EU Clinical Trials Regulation** adopted in 2014, in force since January 2022 [5]. This legislation is under further development by the national Heads of Medicines Agencies (HMA) and the European Medicines Agency (EMA) in their Network Strategy to 2025. As part of this, the fundamental principles of Good Clinical Practice (GCP) will be kept in place but will be more needs-oriented. Unnecessary bureaucracy should be avoided, application procedures simplified and made more transparent.
- **Support from the regulatory authorities:** The Accelerating Clinical Trials in the EU (ACT EU) initiative aims to further solidify the EU as a hub for clinical research, to continue to fund the development of high-quality, safe and efficacious medicinal products whilst at the same time achieving a better integration of clinical research into the European healthcare sector. The deliverables defined in the ACT EU workplan included the rapid establishment of functional, digital platforms and the setting up of the STARS Coordination and Support Action on “Strengthening Training of Academia in Regulatory Science” for the further and continuing education of clinical researchers (Figure 1 (Fig. 1)).
- **Regulatory environment – “lessons learned” from the pandemic towards a future-proof regulatory system:** COVID-19 challenged the EMA whilst bringing about change in equal measure. The main lesson learned from the pandemic is that a maximum of flexibility, agility and cooperation is needed. The rolling review procedure enabled the approval for vaccines etc. to be fast-tracked. Disadvantages include a greater uncertainty about the data, less time to assess the data and a shifting of the assessment of key data and analyses to the post-marketing period. One positive side effect of the pandemic was the “digital push” for point-of-care data.

The key take-home from this lecture was that an innovation-friendly mind-set towards modern patient care is needed.

### 3.2 Benefit assessment of medicinal products

The Scientific Medical Societies play an important role in the integration of new medicinal products into care delivery. Their substantive spectrum of activity extends from basic research, the conduct of clinical trials and the development of clinical practice guidelines to assessments of the added benefit of medicinal products. This lecture focused on:

- **Participation of the scientific medical societies in the process of early benefit assessment of new medicinal products (AMNOG procedure):** The AMNOG procedure, named after the German Medicines Market Reorganization Act (Arzneimittelmarktneuordnungsgesetz), is applied when drug manufacturers set prices and is not primarily a core responsibility of the Scientific Medical Societies. It is their task, however, to ensure that the assessment is evidence-based and patient oriented and to act as a bridge between the assessments by the Federal Joint Committee (G-BA) and the clinical practice guidelines of the Scientific Medical Societies [6]. In the past years, the Scientific Medical Societies have participated in around 90% of all assessment procedures [7]. In 2019, the increasingly pivotal role of investigators and investigation centres was codified in an amendment by virtue of the **German Act for More Safety in the Provision of Medicinal Products (Gesetz für mehr Sicherheit in der Arzneimittelversorgung, GSAV)**. Accordingly, the Scientific Medical Societies are now also involved in the consultations of the Federal Joint Committee (G-BA) in that they prepare expert reports for them.
- **Methodological deficiencies in the assessment procedure:** In key methodological questions, the AMNOG procedure differs from the methodology of clinical practice guidelines in that health technology assessment concepts focused on added benefit and cost effectiveness are at contrast with the concepts of an evidence-based medicine aligned with individual patients. This contrast can lead to discrepancies across assessments and subsequently to uncertainty in care delivery, specifically when it comes to prescribing medicinal products. Their methodologies differ in the formation of subgroups but also in substantive approaches to the assessment of endpoints, such as prolongation of overall survival, prolongation of disease-free or progression-free survival, side effects, quality of life, and other patient-reported outcome parameters.
- **Shortages in medicinal products:** What good are the best concepts and assessments if a medicinal product is not available due to bottlenecks in the supply chain? In the past years, the Scientific Medical Societies have taken on a pivotal role in this area as well. Depending on the Scientific Medical Societies’ assessment as to whether a medicinal product is indispensable or whether there are equally effective alternatives, the more recent statutory regulations will facilitate the importation of medicinal products from abroad.

In conclusion, it is imperative that the Scientific Medical Societies recognize and react to their growing roles. This includes the professionalization and optimization of organizational structures and also mandates a change in the mind-set in each of the disciplines of the Scientific Medical Societies from that of advocacy for their own genuine topics to one of cooperation. 

### 3.3 Clinical trials

Over the past decades, the Coordinating Centres for Clinical Studies (KKS) have played a pivotal role in the substantive and organizational design of clinical trials in Germany [8]:

- **History:** A lack of patient-oriented clinical research in Germany has been identified more than 20 years ago. The KKS Network was established after two tendering rounds held by German Federal Ministry of Education and Research (BMBF). The objective was to create study-related scientific institutes at university medical centres and align them with international standards.
- **Services:** Since its founding, the number of KKS members has grown continuously. In 2022, 25 university medical facilities joined the ranks of this not-for-profit association. For an analysis in 2020, the KKS members had taken on central support tasks in 1,203 trials. Both investigator-initiated trials (IIT) and industry sponsored trials (IST) are supported. The mission of the KKS Network is to support the practical implementation of clinical trials as well as to promote further training and continuing education at the member sites. In 2020, 319 basic training, advanced, refresher and specialist training courses were held at the member sites with over 7,800 attendees.
- **Objectives achieved?** Among the achievements registered are an increase in the quality of clinical trials, the establishment of clinician scientist programmes, funding programmes for clinical trials on the part of the German Research Foundation (DFG) and the German Federal Ministry of Education and Research (BMBF) alongside an increasing evidence-orientation in policymaking. The negative side revealed factors such as there being too few IITs compared to other countries and the fact that funding programmes are being underutilized. This is compounded by a lack of centralised study strategies and a heterogeneity of sites whilst university medical centres are increasingly coming under economic pressure. On top of that, there are additional barriers affecting organizational and substantive issues.

**Figure 2:**
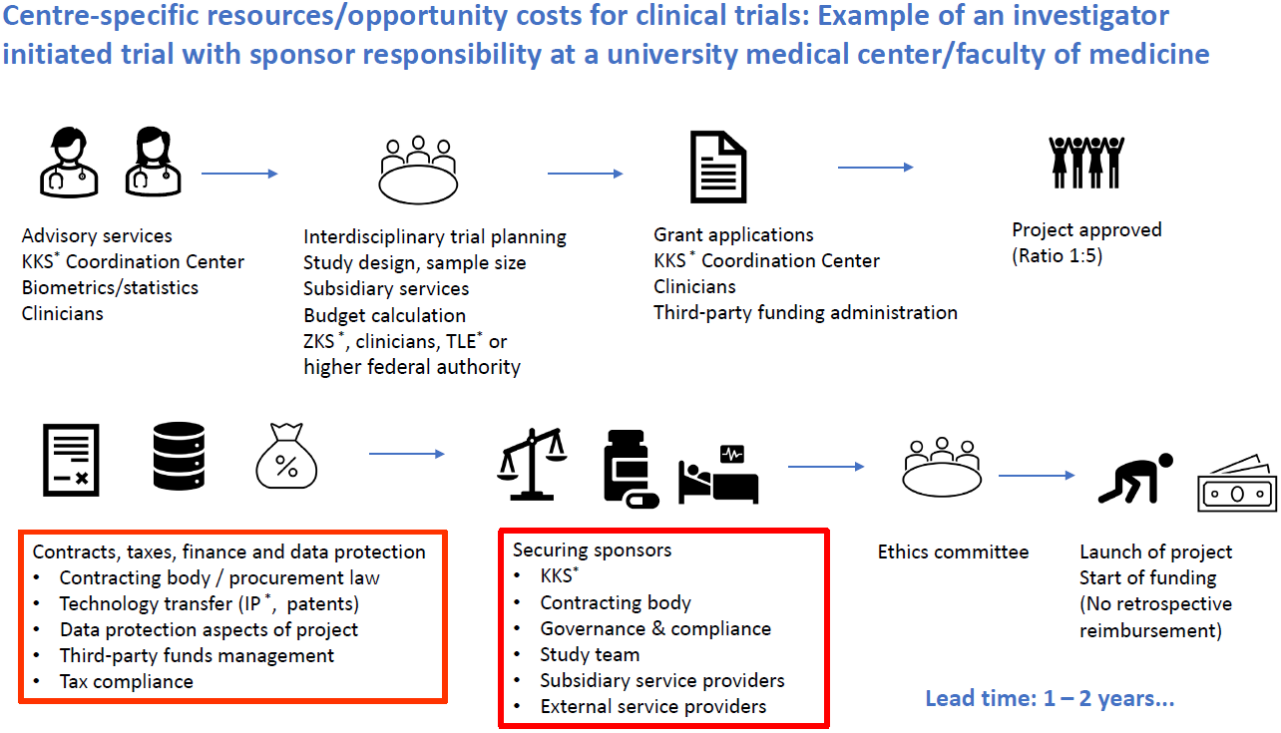
Prototypical sequence for planning and conducting a clinical trial Clinical trials on and with sick or healthy persons constitute the cornerstones of healthcare within the meaning of an evidence-based medicine in constant flux. On the one hand, this applies to the marketing authorisation of medicinal products, medical devices or digital health apps. On the other, this also applies to the development and updating of clinical practice guidelines. This scheme illustrates the complexity involved in planning and conducting such clinical trials. The red boxes indicate the time-consuming processes that would benefit from a reduction in bureaucratic red tape. Legend: BOB=Bundesoberbehörde, higher federal authority, IP=intellectual property; KKS=Coordination Center for Clinical Trials, ZKS=Center for Clinical Trials Tübingen, TLE=Trial Evaluation Center Source: Lecture B. Lang at the Berlin Forum, KKS Network (Association of Academic Coordinating Centres for Clinical Studies in Germany, https://www.kks-netzwerk.de/en/network/about-us/)

In conclusion, it should be stressed that high-quality clinical trials constitute the fundamental building blocks of an evidence landscape from which the entire healthcare system benefits. The prerequisites for this include interdisciplinary teamwork, robust networks and the will to have an evidence-based health and research policy in place (Figure 2 (Fig. 2)).

### 3.4 Recommendations

To achieve a better transfer of evidence to care delivery, the AWMF recommends:

- Close cooperation between national and EU levels
- Integration of evidence-based medicine into clinical trials from the planning and data collection phases down to early and long-term evaluations
- In studies initiated and financed by pharmaceutical industry: participation of the Scientific Medical Societies in the consultations with the regulatory authorities to ensure that marketing authorizations of new medicinal products are informed by the current standard of care
- For all clinical trials in Germany: utilisation of the resources of the KKS to assure the quality of clinical trials and increase the number of patients enrolled in them
- Breaking down overly burdensome bureaucratic hurdles, also when complying with data protection regulations
- Funding of independent clinical trials conducted as investigator-initiated trials (IITs) or health services research
- Creation of a statutory possibility for payers (statutory health insurance) to actively participate in clinical trials

## 4 Clinical practice guidelines as a foundation for a scientifically informed health policy

Another prerequisite for transferring research results to care delivery is the development and regular updating of high-quality clinical practice guidelines alongside the fast availability of guideline statements at the bedside. This was the second area the Berlin Forum 2022 focused on, with the discussion being chaired by Prof. Ina Kopp, M.D.

### 4.1 How guideline work is implemented by the AWMF and its Scientific Medical Societies

In Germany, the responsibility for clinical practice guidelines lies with the Scientific Medical Societies. Since 1995, the AWMF has been coordinating and supporting the initiatives of the Scientific Medical Societies [1], [9] by setting up a quality-assured guideline registry and providing methodological advice through the AWMF Institute for Medical Knowledge Management (AWMF-IMWi) [10]. All clinical practice guideline projects are registered, classified according to their development methodology (S1–S3) and formally reviewed for compliance with the requirements. This includes mandatory conflict of interest management. The Standing Guidelines Commission of the AWMF regularly adapts the underlying AWMF Guidance Manual and Rules for Guideline Development [11]. Thanks to the high level of engagement on the part of the Scientific Medical Societies, this procedure proved its merits during the COVID-19 pandemic. As per April 2022, the AWMF Registry contained as many as 810 published clinical practice guidelines, 17 of which were on COVID-19 [11], [12]. During this process, the AWMF’s COVID-19 Guideline Task Force worked together with the University Medicine Network [13].

The quality of clinical practice guidelines has improved continuously in general, with ever more of them reaching the highest methodological class (S3). Given the stricter methodological requirements, e.g. in relation to systematic literature searches and critical appraisal of included studies, and the growing number of clinical practice guidelines, many Scientific Medical Societies have professionalised their guideline work and have implemented dedicated guideline secretariates and volunteer guideline committees or guideline officers. 

### 4.2 Use of clinical practice guidelines by the Federal Joint Committee (G-BA)

Clinical practice guidelines are not only used by clinicians, but also by institutions of the healthcare sector. The Federal Joint Committee (G-BA) regularly searches guideline recommendations to determine the standard of care, for example for disease management programmes (DMPs), quality assurance measures, appropriate comparator interventions/therapies for new medicinal products as part of early benefit assessments and for non-pharmacological methods and test guidelines. Clinical practice guidelines similarly play a role in helping to structure care delivery areas in different regulatory contexts (e.g. for the “Human resources in psychiatry and psychosomatics guideline” in psychotherapy) or to provide reviews of prevention and therapy recommendations in other countries (e.g. non-invasive prenatal tests). The Federal Joint Committee (G-BA) expects systematically developed, evidence-based clinical practice guidelines of methodologically high quality which assess the benefit and/or examine existing public sources of information (e.g. from the G-BA itself and the Institute for Quality and Efficiency in Healthcare [IQWiG]) and contain recommendations for patient-relevant endpoints. Emphasis is placed on an independence from industry as well as on transparency in terms of funding and any potential conflicts of interest among the guideline group members. Moreover, clinical practice guidelines must be transferable to the German healthcare setting whilst the justification for the derivation of the recommendations must be understandable. What is missing according to the Federal Joint Committee (G-BA) are high-quality clinical practice guidelines on a variety of topics, such as disorders of lipid metabolism in adults and maternity care for pregnant women. For other diseases like neurodermitis or migraine, lower-level clinical practice guidelines are available (S2k) but there are no evidence- and consensus-based S3 guidelines on these diseases. 

### 4.3 Public funding to support clinical practice guideline development

For a long time, clinical practice guidelines were not publicly financed. This all changed when the German Digital Healthcare Act (DVG) entered into force in 2019. Up to 2024, an annual amount of at least 5 million euros is earmarked for guideline projects (Section 92b (2) of the German Social Code Book V [SGB V]) from the innovation fund for healthcare research. In addition, the IQWiG supports clinical practice guideline projects with evidence syntheses on defined PICO questions with up to 2 million euros annually (Section 139 b SGB V). Applications can be submitted for new clinical practice guidelines, but also for updates. In 2020/2021, tender procedures received 62 applications, of which 41 were funded with a total 12.7 million euros [14]. Main topics are annually defined by the German Ministry of Health (BMG), based on submissions agreed in the Guidelines Commission of AWMF. Evidence synthesis by the IQWiG is still in the trial phase, only a few reports have been completed thus far. Also, the evaluated PICO questions address just a small part of the entire spectrum of clinical practice guidelines.

The AWMF welcomes the new funding options for clinical practice guideline projects. Yet, the earmarked funds only cover a small portion of the costs incurred by the stakeholders in the currently more than 100 registered S3 clinical practice guidelines. Indeed, these funding possibilities provide an important incentive to the stakeholders to engage with methodologically high-quality guidelines.

### 4.4 Digitalisation of guideline knowledge

**Figure 3:**
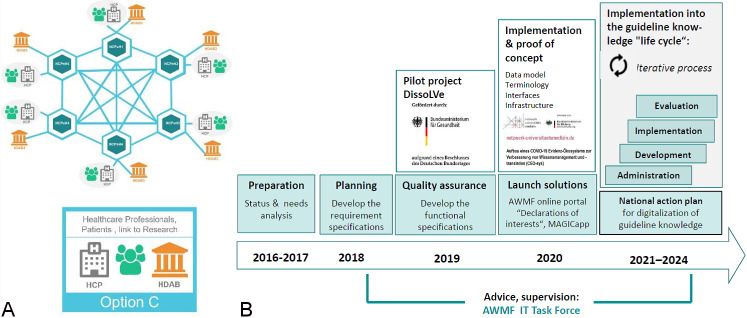
Clinical practice guidelines as foundation for a scientifically informed health policy A: The concept of the European Health Data Space (EHDS): Aimed at the integration of cross-border professional health care services (ePrescriptions, ePatientenakte), citizen-centered services/ (mobile apps for storage and access control of one’s own health data), other services (telehealth, interoperability of infrastructures, patient data sharing, including beyond the EU/EEA), and access to the secondary use of health data by legitimate interest groups (researchers, decision makers etc.). Copyright Figure 3A: European Commission [26] B: The AWMF’s clinical practice guideline project prepares German clinical practice guidelines or their incorporation into the German and European digital European Health Data Space. Copyright Figure 3B: AWMF [15]

The AWMF deems it necessary to digitalise clinical practice guidelines and the AWMF guideline registry so that clinical practice guidelines can be used to translate evidence into care delivery in a forward-looking way [15]. For clinical practice guidelines to be applied at the point of care, easily accessible digital interfaces are required to enable that the shared decision making process can be supported by guideline recommendations. To drive digitalisation, the AWMF Institute for Knowledge Management has conducted pilot projects [16]. Many of the Scientific Medical Societies are actively involved in digitalisation but need structural support. Alongside implementation, digital support can simplify the evaluation of guideline implementation [17]. Evaluations to review the benefit of clinical practice guidelines and expose gaps in research are indispensable and should form the basis of every update (Figure 3 (Fig. 3)).

### 4.5 Recommendations

For a better utilization of evidence in care delivery, the AWMF recommends:

- The integration of the comprehensive digitalisation of clinical practice guidelines into the Data for Health strategy of the German Federal Ministry of Health with the aim of a) creating sustainable digital structures for the quality assurance of clinical practice guidelines, and b) knowledge dissemination at the point of care. Besides the integrated conceptualisation and financing, this also requires legislation to regulate uniform outpatient and inpatient interfaces.
- The continuation of funding for clinical practice guideline projects within the scope of the Innovation Fund beyond 2024 with an additional option of open-topic funding similar to that for healthcare research projects.
- Dedicated support of healthcare research projects on the implementation and evaluation of methodologically high-quality clinical practice guidelines (S3).

## 5 Policy framework and future trends in the European context

In the coming years, the healthcare sector will have to deal with new trends on the European level that impact the delivery of clinical care within national scope to a considerable degree. On the one hand, this applies to legislation governing the maintenance and new development of diagnostic and therapeutic procedures, and, on the other, to trends in the IT sector intended to enable the integrative use of large volumes of data to improve the delivery of healthcare. This was the third area the Berlin Forum 2022 focused on, with the discussion being chaired by Prof. Henning Schliephake, M.D., D.D.S. 

### 5.1 Medical Device Regulation

The **Medical Device Regulation (MDR)**, adopted by the European Parliament and the Council in 2017, entered into force for implementation at the national level in May 2021. One objective of the MDR is to guarantee an improved transparency by means of a Unique Device Identification (UDI) system and the European database on medical devices (EUDAMED) through automatable identification of products. Furthermore, the MDR is intended to enhance patient safety by implementing novel life cycle processes to underpin the clinical assessment and risk management of the medical devices and enable proactive market surveillance by manufacturers. However, numerous hurdles must be overcome when transposing the new regulation into clinical reality at the national level. 

From the today’s perspective, the three greatest challenges to implementation of the MDR are:

1. the clinical trials required for the large number of legacy devices,
2. the considerable rise in costs for recertification and
3. the lack of capacities at the certification agencies termed “notified bodies”.

In principle, the MDR does not impose unfulfillable requirements on the marketability of medical devices when it comes to legacy devices. Indeed, it contains provisions that help ensure continued availability of the products as long as manufacturers can prove that they have initiated the necessary steps in a timely manner. Within this process, transition periods of 3–5 years have been allotted for legacy devices. The German Federal Ministry of Health believes that these periods allow sufficient time for clinical data to be presented for the recertification of legacy devices. Nevertheless, applications for recertification have only been submitted on 10% of all pending legacy products to date. Here, a reticence on the part of the manufacturers is apparent, which is all the more astonishing since grandfathering is limited under the new legislation. 

One of the reasons for this reticence is the high burden complained about by manufacturers, both financially and in terms of tying up personnel to recertify legacy devices under the MDR. The low number of recertifications performed to date may therefore be a barometer that signals an impending thinning-out of the market for legacy devices in subsequent years. The high administrative hurdles seem even more daunting when it comes to orphan devices where the requirement to provide complete clinical evidence poses an obvious problem. With a view to the third hurdle in MDR implementation, the existing bottleneck caused by too few notified bodies is beginning to resolve itself. Their number is anticipated to increase to 44 by the end of 2023. However, the search for a suitable notified body currently still poses a serious problem for many manufacturers. 

In aggregate, the extra regulatory efforts associated with the implementation of the MDR generates a significantly increased demand for resources for manufacturers and creates risks for healthcare delivery due to 1) reduction in product range, 2) potential shortages in the supply of proven medical devices and 3) potential suppression of innovation. 

### 5.2 In Vitro Diagnostic Regulation (IVDR)

The second comprehensive new regulation at the European level is the **In Vitro Diagnostic Regulation (IVDR)** which was supposed to enter into full force in May 2022 after a five-year transition period. Various factors were responsible for the slow implementation of the IVDR by European institutions and companies. Therefore, the EU legislators decided on a more nuanced, deferred application of the regulation with transition periods of up to five years. Notwithstanding, all new diagnostic products being placed on the EU market must comply with the provisions of the IVDR. 

Due to the massive increase in regulatory requirements placed on manufacturers both for the recertification of legacy products as well as for the initial certification of new test kits, there is a risk that this change in the policy framework will lead to a shortage of established IVD tests on the market. This fact – combined with the still too low number of notified bodies and a partial lack of EUDAMED database modules – may jeopardize the supply of essential diagnostic tests for years to come. This particularly applies to the niche tests that only garner small market segments and to products manufactured by smaller companies. 

The IVDR provisions also make it problematical to deal with the numerous non-commercial, laboratory-developed diagnostic tests. Depending on the laboratory’s degree of specialization, self-developed in-vitro diagnostics are used in between 50 and 90% of the assays and are especially indispensable to personalised medicine whilst supplying valuable diagnostic information in early stages of generalised diseases as well. The authorities in the German federal states determine how the interpretation and implementation of the IVDR is transposed for non-commercial laboratory-developed tests.

### 5.3 Legislation regulating the use of registry data

The problems associated with the implementation of MDR and IVDR illustrate how much a transparent and efficient implementation of legislation ultimately relies on the availability of large amounts of data in well-organised registries. In this context, medical data are not only essential for the tracking and quality control of medical devices and diagnostics, but for medical research as well. In particular, access to data derived from care delivery is essential to medical research so that these data can be used to generate hypotheses and promote the development of new diagnostic and therapeutic approaches (secondary use). 

Since 2016, the objective of the **Medical Informatics Initiative (MI-I)** of the German Federal Ministry of Education and Research (BMBF) has been to create regulated, secure, data protection-compliant and supervised access to data and tools based on a trustworthy framework of technologies and rules. As part of this new research infrastructure, core elements of MI-I include the data integration centres (DIC) which are being set up at all university hospitals and networked with each other. At a DIC, all data traces of all patients of the centre are synthesised in a clinical data repository in order to then provide them to researchers on request. National consensus-based standards form an important bulwark for the DIC, both in terms of data (e.g. FHIR profiles, classifications like ICD-10 and ICD-11, terminologies like SNOMED CT) and processes (e.g. regulations governing use, user agreements etc.), which facilitate multi-site projects considerably.

For research use, a Trusted Third Party transfers the initially patient-related clinical data to a research data repository, under defined precautions (including but not limited to pseudonymisation, broad consent, central study registry). During this transfer process, use and access committees (UAC) review the data use application to decide whether compliance with methodological, legal and ethical principles is given. Beyond DICs at university medical centres, the six Digital Progress Hubs Health funded by the German Federal Ministry of Education and Research (BMBF) (2021–2025) are intended to apply concrete medical “use cases” to demonstrate how the MI-I concepts can also be transferred to regional care delivery and intersectoral research.

In this age of the electronic data capture (EDC) of health data in the registries of the statutory health insurance companies, large volumes of routine clinical data are generated that are potentially also valuable for investigating the therapeutic benefit of treatment interventions. Furthermore, the recently adopted regulation on the operation of a cross-national implant registry continues to prescribe the capture of comprehensive data on a large number of various implant types. In conjunction with the aforementioned EUDAMED database, the large quantities of data generated there represent a valuable resource for medical science and clinical research. 

To mitigate the risks of implementing the MDR, the AWMF has drafted concepts for the Scientific Medical Societies and presented them in three statements and at two hearings [18], [19], [20]. Together with the German Society of Surgery, the AWMF is currently focusing the work in its ad hoc committees on exposing supply shortages that impair the quality of patient care at an early stage. Here, registries have been installed that collect data on supply shortages as a potential basis for exemptions pursuant to Article 59 MDR and for overarching considerations. 

### 5.4 Recommendations

For the national implementation of the MDR and IVDR, the AWMF recommends:

- The simplification of recertification of legacy devices. One suitable assessment criterion could be a product’s long anomaly-free lifetime in a registry based on defined quality criteria. These registries should be maintained by the relevant Scientific Medical Societies. The resulting relief afforded the notified bodies of not having to recertify legacy devices would in turn free up capacity for innovation testing.
- The acceleration of market access for innovative medical devices by a “premarket approval process”, accompanied by continual monitoring in order to enable the notified bodies to conduct rolling reviews
- For orphan devices, a certification subject to conditions, e.g., an intensified post-market clinical follow-up (PMCF), setup/use of product registries by the Scientific Medical Societies
- A medically and scientifically sound enforcement of the IVDR with support from the AWMF ad hoc committee on in vitro diagnostics. This is necessary to avoid creating barriers that stifle innovation, especially for innovations by institutions of university medicine.
- The harmonization of data collection in registries and repositories in the context of the Medical Informatics Initiative (MI-I) with respect to structure and terminology, as well as access for data use and evaluation by the medical scientific community whilst keeping in compliance with the statutory provisions governing data protection
- The quality-assured combination of existing and emerging databases for the creation of data structures, which, when subjected to scientific evaluation, can contribute to the expansion of our medical understanding of documented diseases and can be used to apply the appropriate consequences to prevention and treatment
- The preparation for an intersectoral care structure via evidence-based and scientifically verified data
- A more emboldened approach by local ethics committees to their handling of data protection options based on the research exemption provided by the General Data Protection Regulation (GDPR)

## 6 Discussion

Evidence-based medicine should be the driver of patient care. Over the past three decades, this paradigm has increasingly become reality, receiving additional impetus during the COVID-19 pandemic. The prerequisites for fulfilling such a claim are clinical trials conducted as closely as possible to everyday clinical reality so the results can be incorporated quickly into care delivery. The 2022 Berlin Forum highlighted both the positive and negative aspects, juxtaposing the objectives achieved with existing hurdles. The latter must dominate public debate if change is to take place. 

The ongoing changes in approval and availability of medicinal products and medical devices illustrate some of the core problems associated with the bidirectional interactions between patient care and generation of scientific evidence (Table 3 (Tab. 3)).

### 6.1 Bureaucratic hurdles 1

The well-intentioned **EU Clinical Trials Regulation** sets high bureaucratic hurdles for academic developers and other small entities. Networks like KKS offer support, but additional national regulations in Germany hamper progress. Many large international studies complete their recruiting phases long before all German regulatory hurdles have been cleared. The EU has recognised these problems and developed the Network Strategy to 2025. In Germany, too, superfluous hurdles must be eliminated, and processes accelerated with tighter deadlines. 

### 6.2 Bureaucratic hurdles 2

The well-intentioned **Medical Device Regulation** is also proving to be an obstacle, on the one hand for registration of innovations by academic institutions and small and medium-sized enterprises, and on the other hand for availability of legacy devices due to the effort required for their (re-)registration.

### 6.3 Commercial interests

Specifically the area of medicinal products and medical devices is dominated by the commercial interests of globally operating companies. On the positive side, most progress would not happen without the commitment of industry. Once a product has been approved for the German market, the expectations of company’s owners and shareholders are correspondingly high. The preservation of intellectual and economic independence presents a great challenge for the Scientific Medical Societies. Maximum transparency with minimal conflicts of interest is one indispensable prerequisite. 

### 6.4 Independent studies

The strong economic power of industry has almost completely displaced that culture of large publicly financed multicentre trials that still existed in the 1980s. This independent study culture aligned with health care in Germany should be rebuilt.

### 6.5 Health technology assessment versus evidence-based medicine

This antagonism is not a fundamental contraction in terms since there are many methodological aspects that overlap. Nevertheless, the health technology assessment (HTA) approach with the recently reinstated assessment of medicinal products/methods based on quality-adjusted life years (QALYs) is driven by the need to contain health care costs. As was to be expected, the recommendations of clinical practice guidelines diverge substantially from HTA assessments. Clinical practice guidelines are designed to be relevant to patients and appropriate to the point of health care delivery. However, they are only as good as the quality of their data basis, the process of their analysis and their up-to-dateness. This challenge often collides with the highly committed yet still voluntary work performed by guideline developers.

## 7 Conclusion

The Berlin Forum demonstrated the high standards of patient care in Germany, including timely availability of medicinal products and medical devices. But it also made it very clear that the preservation of what has been achieved and further progress mandate changes both in the way evidence is gathered in clinical trials and the way the results are evaluated. These changes should emphasize both medically justified need and patient safety. The recommendations derived in this AWMF position paper are realistic, suitable for broad consensus and should be implemented swiftly.

## Notes

### Acknowledgments

We would like to thank the external lecturers who spoke at the Berlin Forum: Karl Broich, Ulrich M. Gassner, Josef Hecken, Britta Lang and all those who took part in the discussions for their suggestions for this position paper. 

### Competing interests

The authors declare that they have no competing interests.

## Figures and Tables

**Table 1:**
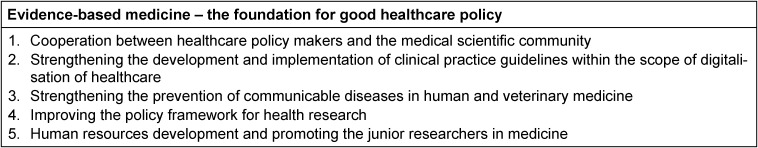
AWMF’s policy demands To accelerate the incorporation of new evidence from medical science into patient care, it is necessary to ensure a good flow of information from the scientific stakeholders to the policy decision-makers. First asserted by the AWMF during the 2017 German parliamentary election, this demand has increasingly been put into practice. Yet in the current legislative period, there is still a need for further action in all five of these topical areas. Source: AWMF [21], [22]

**Table 2:**
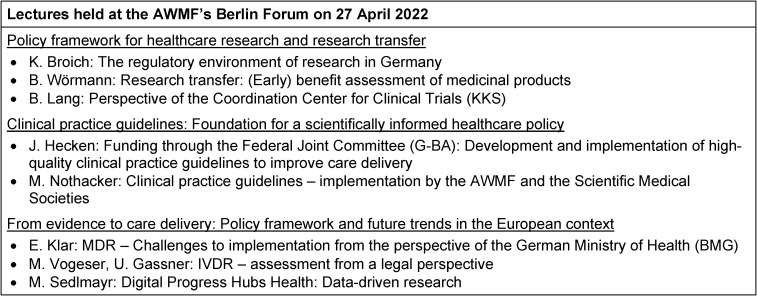
Lectures held at the AWMF’s Berlin Forum on 27 April 2022 Source: AWMF [23]

**Table 3:**
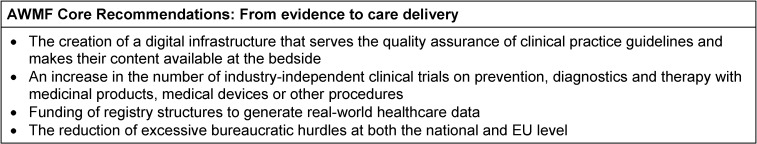
AWMF Core Standards for a better transfer of evidence to care delivery
